# Utilization of medications for neuropathic pain in patients with and without knee and hip osteoarthritis in Sweden: A population-based cohort study

**DOI:** 10.1016/j.ocarto.2026.100856

**Published:** 2026-08-05

**Authors:** Clara Hellberg, Aleksandra Turkiewicz, Kristina Sundquist, Andrea Dell’Isola, Tom Appleyard, Dahai Yu, Geraint Thomas, George Peat, Martin Englund

**Affiliations:** a Clinical Epidemiology Unit, Orthopedics, Department of Clinical Sciences Lund, Lund University, Lund, Sweden; b Center for Primary Health Care Research, University Clinic Primary Care Skåne, Department of Clinical Sciences Malmö, Lund University, Sweden; c Primary Care Centre Versus Arthritis, School of Medicine, Keele University, Staffordshire, UK; d Centre for Applied Health & Social Care Research (CARe), School of Health and Social Care, Sheffield Hallam University, Sheffield, UK

**Keywords:** Osteoarthritis, Knee OA, Hip OA, Pain, Medications

## Abstract

**Objective:**

Use of neuropathic pain medications is increasing in OA patients, but it is unclear if this is driven by OA-specific use or presence of comorbidities. We aimed to investigate the use of neuropathic pain medications attributable to knee and hip OA pain.

**Design:**

We included all residents aged ≥35 years of the Skåne region, Sweden, in 2018. After exclusions, 645,991 individuals remained, where 81,606 had diagnosed knee and/or hip OA. We compared prevalence of dispensed neuropathic pain medications between those with and without OA using logistic regression (odds ratios approximated risk ratios [RR]), adjusting for comorbidities associated with use of such medications. We interpreted any higher prevalence remaining after adjustment as being compatible with possible OA-related use. Due to interaction effect, males and females were studied separately.

**Results:**

Among OA females, crude prevalence of dispensed neuropathic pain medications was highest among 45-54-year-olds (4.0%), with a decreasing prevalence toward 75-84-year-olds (2.3%) compared to 1.5% in non-OA females. After adjusting for comorbidities, the RR for females with vs without OA was 1.4 (95% CI 1.3–1.6) at age 50 and 1.0 (95% CI 0.9–1.1) at age 80. Males had similar prevalence of use across ages, 1.5% in persons with OA and 0.8% in persons without. After adjustment for comorbidities there was no association between OA and neuropathic medication dispensations (RR 1.0; 95% CI 0.9–1.1).

**Conclusion:**

In particular, middle-aged OA females may use neuropathic pain medications for treatment of OA-related pain, as increased prescribing remained after adjusting for relevant comorbidities.

## 1 Introduction

Nociceptive pain, which results from actual or pending damage to non-neural tissue and mediated by activation of nociceptors, is the most common and well-studied type of pain in patients with osteoarthritis (OA). However, in recent years, the recognition of a multifactorial pain pathogenesis in OA has emerged, including nociplastic pain, arising from altered nociception, and neuropathic pain, associated with structural nerve damage, where 8.2% of knee OA patients have been suggested to have a potential neuropathic pain component [1]. Previous studies have provided moderate-quality evidence in support of the effect of duloxetine, one of the medications used for neuropathic pain in knee OA [2]. Several clinical practice guidelines including Osteoarthritis Research Society International (OARSI), American College of Rheumatology, and The Royal Australian College of General Practitioners, have consequently issued conditional recommendations for the use of duloxetine in knee OA, particularly in cases with widespread and persistent pain [3,4]. In contrast, the quality of evidence regarding the effect of duloxetine on hip OA pain is insufficient, and the OARSI conditionally recommends against its use in hip OA [5].

There are reports of a global increase in prescribed neuropathic pain medications in recent years, both in the general population [6] and in patients with OA [7]. However, some of these medications, such as gabapentinoids, have been associated with risk of addiction and serious adverse events, warranting caution in their prescription [6,7]. At the same time, treatment options for OA pain are limited, which may lead to prescribing medications not formally recommended for OA pain, e.g., in patients with contraindications for NSAID use [8] or when opioid use is to be avoided.

The rising prescription rates of neuropathic pain medications, coupled with rising concerns regarding the need for caution in their use, highlight the need to assess and monitor the usage of these medications in OA patients. Furthermore, although recent studies suggest a potential therapeutic effect, they are still only conditionally, if at all, recommended in the major treatment guidelines [9]. Additionally, these medications have multiple indications beyond pain management, including the treatment of epilepsy and depression [10,11], as well as off-label applications including insomnia and pruritus [12], meaning that a prescription to a patient with OA may not necessarily be attributable to OA-related pain. Therefore, it is important to gain new knowledge of their use in patients with OA and specifically for OA pain management.

Thus, our study aimed to:

- estimate the overall prevalence of prescribed neuropathic pain medications among patients with knee and/or hip OA compared to those without, irrespective of comorbidities.
- estimate the use of prescribed neuropathic pain medications specifically for OA-related pain in the knee and/or hip by accounting for comorbidities.
- examine potential differences in prescribing between patients with knee versus hip OA, as well as variations across sex and age groups.

## 2 Materials and methods

### 2.1 Study population

**Fig. 1:**
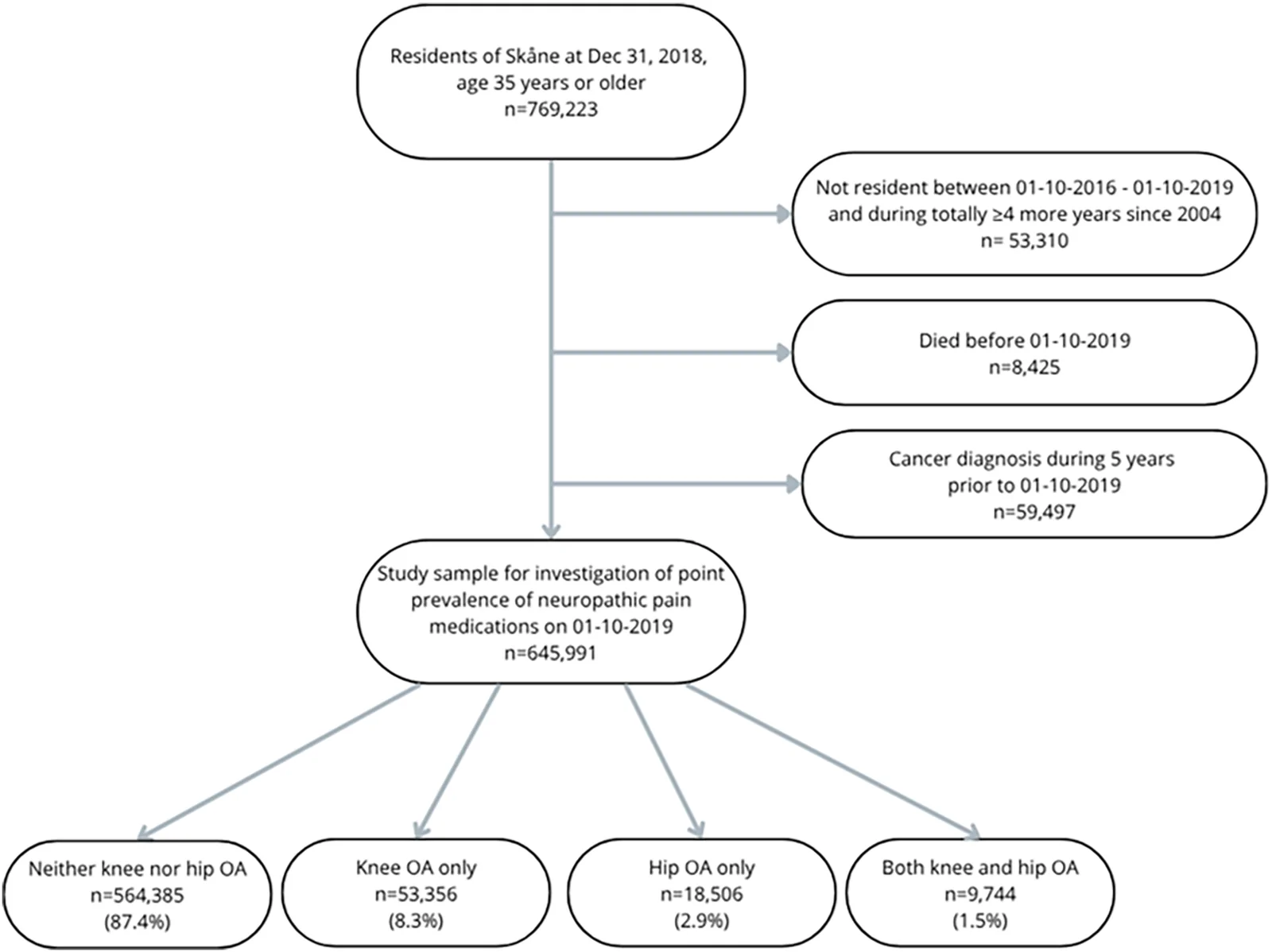
Flowchart of inclusion of study sample and distribution of individuals with or without OA.

We aimed to estimate the neuropathic pain medication use as of October 1, 2019. Therefore, we extracted data for all 769,223 individuals aged ≥35 years, residing in the Skåne region, Sweden, as of December 31, 2018, assuming no relocation from the region during 2019, as residential information is provided annually. Furthermore, we required being resident in the region during 2016–2019 and to have at least 4 more years of prior health data available, to ensure the capture of OA diagnoses and potential comorbidities. Furthermore, patients who died before October 1, 2019 (the date of prevalence estimation) and patients with a cancer diagnosis registered in the Skåne Healthcare Register [13] between September 30, 2014, and September 30, 2019, were excluded, as a cancer diagnosis could obscure or lead to underreporting of other comorbidities, particularly during its more acute phase (a list of cancer diagnoses was compiled based on previous work by Thorlund et al. [14]). Of the remaining 645,991 individuals, we identified all patients with at least one registered ICD-10 code for OA in the knee (M17) or hip (M16) from a registered physician's visit between January 1, 2004, and September 30, 2018, in the Skåne Healthcare Register. Patients with OA in the knee and/or hip, hereafter referred to only as ‘OA’ for simplicity, were classified as exposed, while those without OA (i.e. without knee and/or hip OA) were classified as unexposed. Information on population characteristics and socioeconomic variables was extracted from the registers held by Statistics Sweden. We did not exclude patients with total knee or hip replacement because we could not rule out that the patients were using neuropathic pain medications due to OA in the other hip or knee joint (Fig. 1).

### 2.2 Outcome

Prescribed and dispensed neuropathic pain medications of amitriptyline (ATC code N06AA09), duloxetine (ATC code N06AX21), gabapentin (ATC code N03AX12) or pregabalin (ATC code N03AX16) for all individuals with or without OA were extracted from the Swedish Prescribed Drug Register for the year 2019. In Sweden, treatment with neuropathic pain medications requires a physician's prescription. Consequently, the register captures dispensed prescriptions for these medications, including prescriptions that may be for on-label or off-label use. However, it does not record the clinical indication for each prescription and cannot confirm whether a dispensed medication was consumed.

For the estimation of point prevalence of use of any of these neuropathic pain medications, an individual was categorized as a user on October 1, 2019, when this day was included in the interval between the dispensing date and the theoretical end date of the prescription. The theoretical end date of the prescription was calculated as the dispensing date plus the number of total daily doses dispensed (filled defined daily dose). If there were multiple active dispensations of the same medication on October 1, the one with the closest preceding dispensation date to the index date was chosen. In the case of multiple dispensations of the same medication on the same day, one was chosen randomly.

### 2.3 Comorbidities

ICD-10 codes for comorbidities associated with treatment of the included neuropathic pain medications were extracted from the Skåne Healthcare Register between September 30, 2016, to September 30, 2019 (i.e. 3 years prior to the index date). The Skåne Healthcare Register contains data on diagnoses, including both the primary reason for consultation and relevant comorbidities, assigned at physician visits within Region Skåne, in primary care as well as in both outpatient secondary care and inpatient hospital care. At each physician visit, the patient's personal identification number and the date of the visit are also recorded.

The list of comorbidity diagnoses was compiled through a combination of FDA-approved indications, reporting of off-label use [12,[15], [16], [17]], and Swedish drug treatment guidelines [10,11,18,19] for these medications (Appendix 1, Appendix 2).

### 2.4 Procedure, statistical analysis

The point prevalence of use of neuropathic pain medications was determined on October 1, 2019. This date was selected a priori as it fell in the middle of the fall semester, avoided school breaks, and occurred in the middle of the week (Tuesday), providing a representative time point for analysis. Prevalence proportions of neuropathic pain medication use with 95% confidence intervals (CI) were reported for persons with or without OA, by age group (in 10-years intervals), and sex (female/male was defined according to each person's Swedish personal identity number).

Based on the results from descriptive analyses above, we used a logistic regression model separately for males and females to estimate the use of neuropathic pain medications in knee and hip OA patients compared to individuals without. The model was adjusted for demographics: age (continuous), highest educational level reached (categories), if born in Sweden (yes/no), and if living in an urban area (yes/no). Country of birth was missing for 0.02% and education for 1.14% of the cohort, and persons with missing data were excluded from the adjusted analyses. Additionally, in a later step, we adjusted for comorbidities registered within the 3-year period prior to the index date that could have a prescription of a neuropathic pain medication, e.g. painful diabetic neuropathy, depressive disorder and epilepsy (Table 1 and Appendix 2). The analyses were adjusted for in two stages. The first regression model included adjustment for demographics and the second included adjustment for demographics and comorbidities associated with neuropathic pain medications. By adjusting for comorbidities that may represent indications for the use of neuropathic pain medications, we aim to better identify the proportion of patients with OA who may be using these medications specifically for OA-related pain. Given the low prevalence of the outcome, odds ratios from the logistic regression model can be interpreted as risk ratios (RR). Furthermore, we estimated differences in proportions (i.e. risk differences) with 95% CI from the fitted regression models using command *margins*. We also performed sensitivity analyses where patients were classified as having OA without joint replacement, or as having OA and joint replacement in at least one joint, to examine whether knee or hip replacement surgeries influenced the results. StataNow 18_5 was used for all analyses.

**Table 1:** Population characteristics, socioeconomic variables, and prevalence of comorbidities for individuals with and without OA.

	No OA	Knee and/or hip OA	Knee OA only	Hip OA only	Knee and hip OA
Sample size	564,385 (87.4%)	81,606 (12.6%)	53,356 (8.3%)	18,506 (2.9%)	9744 (1.5%)
Age (years), mean (SD)	56 (14)	69 (12)	68 (12)	71 (12)	75 (10)
Male sex	280,377 (49.7%)	33,240 (40.7%)	22,043 (41.3%)	7812 (42.2%)	3385 (34.7%)
Age groups (years)					
35-44	144,401 (25.6%)	2019 (2.5%)	1548 (2.9%)	444 (2.4%)	27 (0.3%)
45-54	149,447 (26.5%)	8445 (10.3%)	6527 (12.2%)	1574 (8.5%)	344 (3.5%)
55-64	115,750 (20.5%)	17,071 (20.9%)	12,749 (23.9%)	3100 (16.8%)	1222 (12.5%)
65-74	92,619 (16.4%)	25,303 (31.0%)	16,457 (30.8%)	5814 (31.4%)	3032 (31.1%)
74-84	46,297 (8.2%)	20,110 (24.6%)	11,482 (21.5%)	5185 (28.0%)	3443 (35.3%)
85+	15,871 (2.8%)	8658 (10.6%)	4593 (8.6%)	2389 (12.9%)	1676 (17.2%)
Highest educational level reached, n (%)					
Education up to 9 years	95,905 (17.2%)	23,089 (28.6%)	14,595 (27.7%)	5273 (28.7%)	3221 (33.3%)
Education 10–12 years	239,079 (42.9%)	35,879 (44.4%)	23,912 (45.3%)	7798 (42.5%)	4169 (43.1%)
Education 13–14 years	78,799 (14.1%)	9578 (11.9%)	6316 (12.0%)	2239 (12.2%)	1023 (10.6%)
Education 15+ years	144,111 (25.8%)	12,204 (15.1%)	7907 (15.0%)	3047 (16.6%)	1250 (12.9%)
Born in Sweden	442,023 (78.3%)	68,822 (84.3%)	44,126 (82.7%)	16,230 (87.7%)	8466 (86.9%)
Lives in urban area	271,560 (48.1%)	34,995 (42.9%)	23,365 (43.8%)	7692 (41.6%)	3938 (40.4%)
Comorbidities, n (%)	. (.)	. (.)	. (.)	. (.)	. (.)
Arthritis, other	12,759 (2.3%)	4630 (5.7%)	2937 (5.5%)	985 (5.3%)	708 (7.3%)
Back pain, most common; including disc hernia, sciatica and back pain unspecified	68,657 (12.2%)	19,036 (23.3%)	11,549 (21.6%)	4532 (24.5%)	2955 (30.3%)
Back pain, other	12,365 (2.2%)	5799 (7.1%)	3185 (6.0%)	1480 (8.0%)	1134 (11.6%)
Chronic pain	26,816 (4.8%)	33,653 (5.2%)	4490 (8.4%)	1399 (7.6%)	948 (9.7%)
Diabetes mellitus with complications	19,848 (3.5%)	6494 (8.0%)	4112 (7.7%)	1441 (7.8%)	941 (9.7%)
Headache	35,703 (6.3%)	6130 (7.5%)	4104 (7.7%)	1271 (6.9%)	755 (7.7%)
Joint disease, other	34,263 (6.1%)	13,639 (16.7%)	8967 (16.8%)	2713 (14.7%)	1959 (20.1%)
Neurological disease	22,266 (3.9%)	6929 (8.5%)	4087 (7.7%)	1677 (9.1%)	1165 (12.0%)
Neuropathy	28,116 (5.0%)	7886 (9.7%)	4964 (9.3%)	1678 (9.1%)	1244 (12.8%)
OA in other joints^a^	19,993 (3.5%)	14,906 (18.3%)	8979 (16.8%)	3059 (16.5%)	2868 (29.4%)
Other diseases	26,067 (4.6%)	5569 (6.8%)	3682 (6.9%)	1140 (6.2%)	747 (7.7%)
Pain or discomfort, unspecified	63,324 (11.2%)	19,133 (23.4%)	12,125 (22.7%)	4192 (22.7%)	2816 (28.9%)
Psychiatric disease, most common; including depression, anxiety, stress	115,888 (20.5%)	23,017 (28.2%)	14,870 (27.9%)	5063 (27.4%)	3084 (31.7%)
Psychiatric disease, other	35,403 (6.3%)	5555 (6.8%)	3623 (6.8%)	1265 (6.8%)	667 (6.8%)
Soft tissue disease, other	56,254 (10.0%)	17,301 (21.2%)	11,156 (20.9%)	3569 (19.3%)	2576 (26.4%)
Ulcers	8458 (1.5%)	3097 (3.8%)	1746 (3.3%)	798 (4.3%)	553 (5.7%)

a Not included in the regression analyses as it was considered part of generalized OA, potentially also encompassing knee and hip OA.

### 2.5 Ethical approval

The use of Swedish register data was approved by the Swedish Ethical Review Authority, Lund, (Dnr 2011_432 with amendment Dnr 2014_276 and Dnr 2018_233).

## 3 Results

### 3.1 Descriptive data - overall use of neuropathic pain medications in individuals with or without OA

Out of the original cohort of 769,223 individuals residing in Skåne as of December 31, 2018, a total of 645,991 individuals remained after the exclusion criteria were applied. Among these, 81,606 (12.7%) individuals had OA in the knee, hip, or both knee and hip, with the distribution further detailed in the flowchart of inclusion (Fig. 1). Most OA patients were female (59.3%), compared to half of the individuals without OA (50.3%). Additionally, OA patients were older (mean age 69 years; standard deviation 12), compared to the group without OA (mean age 56 years; standard deviation 14) (Table 1).

**Fig. 2:**
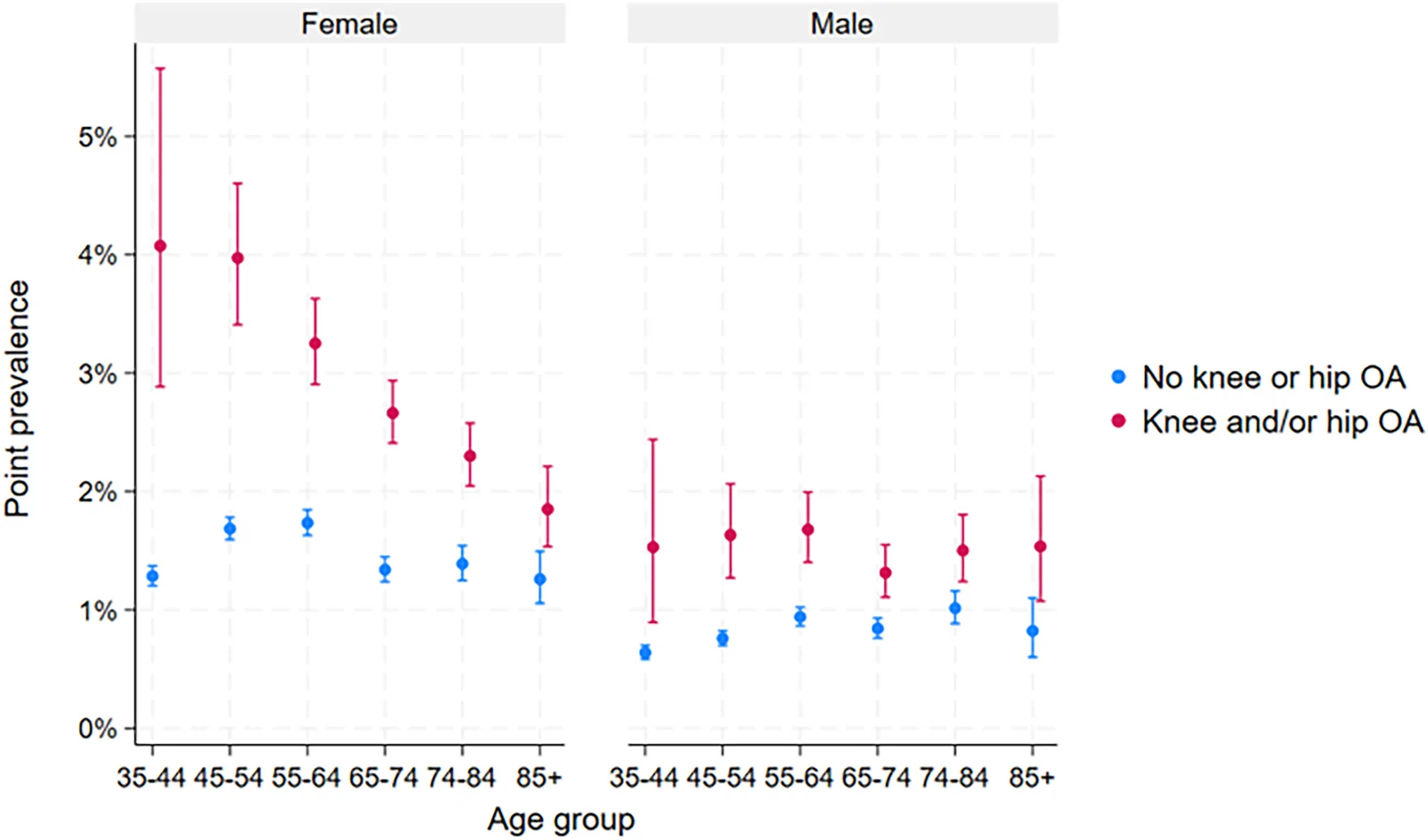
Prevalence of any dispensed neuropathic pain medication with 95% confidence interval in individuals with or without OA for each age group, stratified by sex.

The point prevalence of dispensed neuropathic pain medications, i.e. the use of these medications, was higher in patients with OA, 2.22% (95% CI 2.12, 2.33), compared to individuals without, 1.15% (95% CI 1.12, 1.18) and also higher in those with any comorbidity associated with the treatment (Appendix 3). The use was slightly higher in individuals with both knee and hip OA compared to those with either knee or hip OA alone, with similar proportions observed between the latter two groups (Table 2). Furthermore, the use of neuropathic pain medications varied considerably by sex, where the prevalence among male OA patients remained relatively stable across age groups, 1.50% (95% CI 1.37, 1.64). However, middle-aged females had a higher use compared to more elderly females; e.g., prevalence was 3.97% (95% CI 3.41, 4.60) for the 45-54-year-olds while it was 2.30% (95% CI 2.05, 2.58) for the 75-84-year-olds. In individuals without OA, the use was quite stable across age groups for both sexes, 0.80% (95% CI 0.77, 0.83) for males, and for the females the prevalence of use was 1.69% (95% CI 1.59, 1.78) and 1.39% (95% CI 1.25, 1.54) for the age groups 45–54 and 75–84 years, respectively (Fig. 2). Furthermore, this pattern of use remained consistent when analyzing each OA group (knee, hip, or both knee and hip OA), separately (Appendix 4).

**Table 2:** Prevalence of any dispensed neuropathic pain medication across OA groups (knee, hip, both knee and hip, and knee and/or hip OA) and individuals without OA, in total and stratified by sex, with its 95% confidence intervals (CI).

Prevalence of any neuropathic pain medication									
	Both sexes			Males			Females		
	Total number	Prevalence (%)	95% CI	Total number	Prevalence (%)	95% CI	Total number	Prevalence (%)	95% CI
Knee and/or hip OA	81,606	1814 (2.22)	2.12–2.33	33,240	499 (1.50)	1.37–1.64	48,366	1315 (2.72)	2.58–2.87
Knee OA only	53,356	1144 (2.14)	2.02–2.27	22,043	326 (1.48)	1.32–1.65	31,313	818 (2.61)	2.44–2.80
Hip OA only	18,506	397 (2.15)	1.94–2.36	7812	103 (1.32)	1.08–1.60	10,694	294 (2.75)	2.45–3.08
Hip and knee OA	9744	273 (2.80)	2.48–3.15	3385	70 (2.07)	1.62–2.61	6359	203 (3.19)	2.77–3.65
No knee or hip OA	564,385	6489 (1.15)	1.12–1.18	280,377	2242 (0.80)	0.77–0.83	284,008	4247 (1.50)	1.45–1.54

The most frequently used neuropathic pain medication for OA patients was gabapentin, 0.76% (95% CI 0.70, 0.82), whilst duloxetine was the most common among patients without OA, 0.40% (95% CI 0.38, 0.42).

Among patients with knee OA only, 18.9% (95% CI 18.6, 19.3) had undergone knee replacement, while 44.8% (95% CI 44.0, 45.5) of those with hip OA only had undergone hip replacement.

### 3.2 Comparison of overall use of neuropathic pain medications between individuals with or without OA

In females, the difference in OA-related outcomes were influenced by age, necessitating the inclusion of age interactions in the analysis. Therefore, separate regression models were fitted for the sexes.

#### 3.2.1 Results for males

In the logistic regression model adjusting for demographics (age, sex, educational status, and if born in Sweden) the RR of using a neuropathic pain medication was 1.7 (95% CI 1.5, 1.8) in males with OA compared to those without. However, after adjusting for comorbidities (list of comorbidities in Table 1 and Appendix 2), the RR was 1.0 (95% CI 0.9, 1.1). Similarly, the risk difference decreased from 0.53% (95% CI 0.40, 0.66) to −0.01% (95% CI -0.10, 0.08) between males with and without OA after adjusting for comorbidities.

Furthermore, the pattern of use remained consistent regardless of OA site (knee, hip, both knee and hip OA, or no OA; Appendix 5) and when examining the use in OA males with knee or hip replacement (Appendix 6).

#### 3.2.2 Results for females

In females, interactions between OA and age were included in the analysis, with an interaction RR of 0.84 (95% CI 0.80, 0.88) when adjusting for demographics and of 0.88 (95% CI 0.84, 0.93) when also adjusting for comorbidities per 10 years of age difference. The risk of using a neuropathic pain medication was highest among middle-aged females with OA compared to females of the same age groups without OA, with gradually decreasing RR and risk difference in the older age groups. Notably for females, the higher risk of using a neuropathic pain medication persisted in the middle age groups, after adjusting for comorbidities. In contrast, for the oldest age groups, the RR approached 1 and the risk difference zero after adjusting for comorbidities (Table 3 and Appendix 7).

**Table 3:** Risk ratio and risk difference with its 95% confidence intervals (CI) of a dispensed neuropathic pain medication between females with and without OA adjusting for demographics and for demographics + comorbidities. Presented by age group (age in midpoints for the age group intervals in descriptive table).

	Adjusted for demographics		Adjusted for demographics and comorbidities	
Age (years)	Risk ratio	95% CI	Risk ratio	95% CI
40	3.0	2.6–3.5	1.6	1.4–1.9
50	2.5	2.3–2.8	1.4	1.3–1.6
60	2.1	2.0–2.3	1.3	1.2–1.4
70	1.8	1.7–1.9	1.1	1.0–1.2
80	1.5	1.4–1.7	1.0	0.9–1.1
90	1.3	1.1–1.5	0.9	0.8–1.0
	Risk difference %	95% CI	Risk difference %	95% CI
40	3.20	2.56–3.84	1.04	0.66–1.42
50	2.32	1.95–2.69	0.68	0.45–0.92
60	1.62	1.42–1.83	0.40	0.26–0.54
70	1.08	0.93–1.23	0.17	0.06–0.28
80	0.65	0.49–0.82	−0.01	−0.14–0.12
90	0.33	0.13–0.53	−0.15	−0.31–0.00

Demographics: age, sex, educational status, and if born in Sweden.

Comorbidities: see Table 1.

**Fig. 3:**
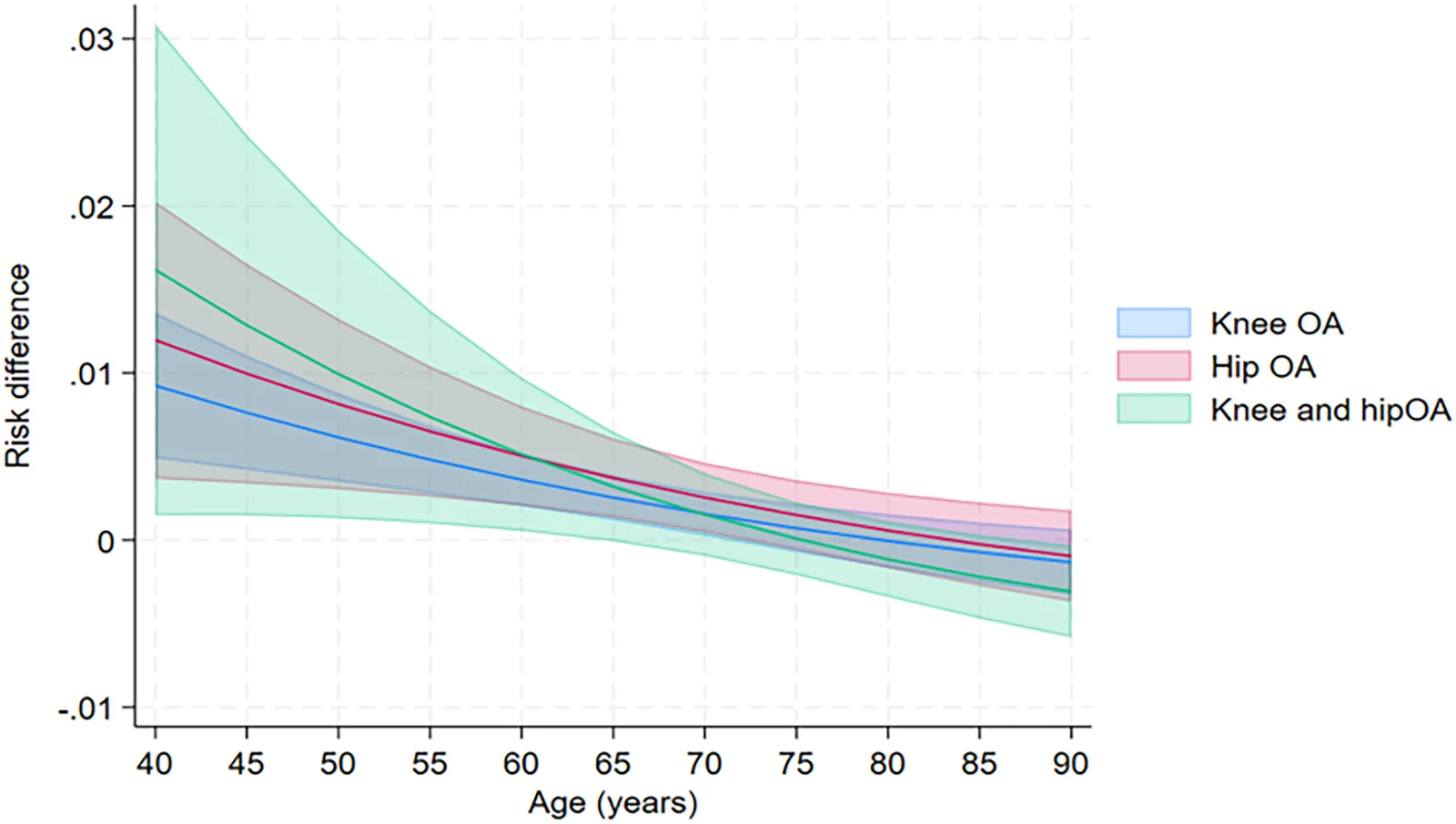
Risk difference with 95% CI after adjusting for demographics and comorbidities, presented by age, for groups of OA (knee, hip and knee and hip OA), between females with and without OA.

This pattern of use remained consistent across all OA sites, with middle-aged females having a higher risk of using a neuropathic pain medication compared to the older ones, where the higher use persisted among middle-aged females after adjusting for comorbidities. Notably, the CI for use of neuropathic pain medications across the different OA subgroups largely overlapped (Fig. 3 and Appendix 8).

In the analysis of use of neuropathic pain medications in female OA patients with or without joint replacement, an interaction effect was observed for females with OA but without knee and/or hip replacement compared to females without OA (interaction RR 0.89; 95% CI 0.84, 0.95), as well as for females with OA with knee and/or hip replacement compared to females without OA (interaction RR 0.80; 95% CI 0.72, 0.88). The pattern of neuropathic use remained consistent as for previous analyses in females, showing a persistently elevated risk of use among middle-aged females with or without joint replacement after adjusting for comorbidities (Appendix 9).

## 4 Discussion

Using a comprehensive population-based data set, we found that the prevalence of neuropathic pain medication use was higher among patients with OA (in the knee and/or hip) compared to a non-OA population. However, after adjusting for comorbidities associated with use of neuropathic pain medications, to assess the likely use for OA pain management specifically, the risk was no longer elevated among the males and older females with OA. Interestingly, though, a higher prevalence of dispensed neuropathic pain medications persisted among middle-aged females with OA, even after adjusting for comorbidities. Furthermore, we found similar levels of neuropathic pain medication use irrespective of having OA in the knee or hip for both males and females.

Our findings suggest that male OA patients primarily used the medications for reasons other than their OA-related pain and similar results were observed for the older females with OA. However, a noteworthy finding was that middle-aged females with OA may often use these medications for OA-related pain. Avoiding potentially harmful treatments, such as opioids, is particularly important in a younger population. This, combined with the limited pharmacological alternatives for the treatment of OA, may potentially explain why physicians explore other, less well-established treatments in this patient category. However, the same pattern was not seen in middle-aged male OA patients. Future research investigating potential differences in treatment effects between males and females, or a potentially higher prevalence of neuropathic pain among females, may provide further insights.

Although the prescribing of medications for neuropathic pain has been reported to increase in patients with OA, few studies have specifically examined their use for OA-related pain. However, one such study estimated that approximately 9% of OA patients were prescribed gabapentinoids for OA-related pain. Furthermore, the authors reported that younger females with OA were the most likely to receive gabapentinoid prescriptions, although the specific indication for these prescriptions remained unclear [20]. Another study reported a lower, yet still notable, use of neuropathic pain medications in a subgroup of OA patients without comorbidities typically associated with such medications, compared to the broader OA population. For the broader OA population, female sex was associated with a higher likelihood of being prescribed antidepressants or anticonvulsants. However, in contrast to our findings, older age was also identified as a risk factor for such prescribing [7].

In our study, we included a comprehensive list of comorbidities, which may have accounted for residual confounding for males and older females with OA, compared to other studies. Furthermore, our analysis focused specifically on patients with knee and hip OA, utilizing a comprehensive data set from a well-defined geographical area and register resources of both exposure and outcome with high validity [21]. We encourage future research to replicate similar analyses in other populations and settings. Such efforts would help validate our findings and further investigate the potential factors contributing to the higher use of neuropathic pain medications among middle-aged females with knee and hip OA.

This study is subject to certain important limitations. First, since we aimed to estimate the use of neuropathic pain medications specifically for OA-related pain, we adopted a conservative approach by including a broad range of comorbid conditions, thereby accepting potential over-adjustment rather than the risk of omitting relevant confounding diagnoses. By applying such a comprehensive list of comorbidities, we hope to have minimized the likelihood that the higher prescribing observed among patients with OA can be explained by comorbidities. However, we cannot entirely exclude the presence of residual confounding or unidentified interaction effects, potentially particularly relevant among middle-aged females, which may contribute to their persistently elevated use after adjustment. Conversely, this approach may also have led to an underestimation in use of neuropathic pain medications specifically for OA pain, if the drug was prescribed both for a comorbidity and OA pain.

Furthermore, we included only patients with a knee and/or hip OA diagnosis recorded during a physician's visit. As a result, patients who have not sought medical care or who only consulted a physiotherapist or other healthcare professionals are not represented in our sample, which accounts for approximately 33% of patients with knee OA [22]. As we have likely missed patients with milder forms of symptomatic OA, the proportion of patients using neuropathic pain medications in our study might be higher than would be seen in a cohort where all severity levels of OA are included. In Sweden, the diagnosis of knee and hip OA is recommended to be made clinically based on patient history, symptoms, and physical examination of the joint [23]. Hence, it can be expected that the majority of patients recorded with a diagnosis of knee or hip OA in Sweden have symptomatic OA. However, we do not know how many of these patients also exhibited radiographic changes typically consistent with OA.

In conclusion, we found a higher use of neuropathic pain medications among patients with knee and/or hip OA compared to individuals without. Middle-aged females seem to be using neuropathic pain medications for OA-related pain more than others.

## Author contributions

All authors were involved in the conceptualization and design of the study. The first author, CH, was responsible for writing the original draft. AT and CH conducted the statistical analyses. All authors contributed to the review and editing of the manuscript and have given final approval of the manuscript to be submitted. ME served a supervisory role in the project. CH (clara.hellberg@med.lu.se) takes responsibility for integrity of the work.

## Declaration of Generative AI and AI-assisted technologies in the writing process

During the preparation of this work the author(s) used ChatGPT to assist with the translation of briefer sections from Swedish to English, as well as to improve English grammar in other sections originally written by us in English. After using this tool/service, the author(s) reviewed and edited the content as needed and take(s) full responsibility for the content of the publication.

## Role of the funding source

The study was financed by Swedish governmental funding of clinical research (ALF), through which CH used the funding to offset salary costs for the time dedicated to working on the manuscript. Additional sources of funding were Region Skåne (SUS Donations), Sweden; The 10.13039/501100004359Swedish Research Council, Sweden; Österlund Foundation, Sweden; Gustaf V 80-Year Birthday Foundation, Sweden; the 10.13039/501100007949Swedish Rheumatism Association, Sweden; the Foundation for People with Movement Disability in Skåne, Sweden; the 10.13039/501100006075Greta and Johan Kock Foundation, Sweden; and 10.13039/501100022244NIHR Applied Research Collaboration West Midlands, United Kingdom. None of the latter have been used specifically for funding this article and no funder have had influence on study design, analysis, decision regarding publication or the like.

## Competing interest statement

ME reports past consultancy for Novo Nordisk, Genascence, Grünenthal Sweden AB and Key2Compliance AB. AT reports receiving fees as Associate Editor for statistics for Osteoarthritis and Cartilage. AD reports involvement as Chair of OARSI multimorbidity discussion group.

## Figures and Tables

**Table:** 

Swedish national drug treatment guidelines according to FASS.se			
Gabapentin	Pregabalin	Amitriptyline	Duloxetine
Peripheral neuropathic pain such as diabetic neuropathy and postherpetic neuropathy.	Peripheral and central neuropathic pain	Major depressive disorder	Major depressive disorder
Monotherapy or adjunctive therapy in treatment of partial epileptic seizures with or without secondary generalization.	Adjunctive therapy for partial-onset epileptic seizures, with or without secondary generalization	Neuropathic pain	Generalized anxiety disorder
Restless leg syndrome	Generalized anxiety disorder	Migraine and tension headache prophylaxis	Neuropathic pain associated with diabetic peripheral neuropathy
			Stress urinary incontinence

FDA approved indications			
Gabapentin	Pregabalin	Amitriptyline	Duloxetine
Postherpetic neuralgia	Neuropathic pain associated with diabetic peripheral neuropathy	Major depressive disorder	Major depressive disorder
Adjunctive therapy in treatment of partial epileptic seizures with or without secondary generalization.	Neuropathic pain associated with spinal cord injury		Generalized anxiety disorder
Restless leg syndrome	Postherpetic neuralgia		Chronic musculoskeletal pain
	Adjunctive therapy for partial-onset epileptic seizures		Neuropathic pain associated with diabetic peripheral neuropathy
	Fibromyalgia		Fibromyalgia

Non-FDA approved indications (off-label use)			
Gabapentin	Pregabalin	Amitriptyline	Duloxetine
Depressive and mood disorders	Generalized anxiety disorder	Anxiety	Stress urinary incontinence
Anxiety and social phobia	Social anxiety disorder	Irritable bowel syndrome	
Post-traumatic stress disorder	Trigeminal neuralgia	Post-traumatic stress disorder	
Insomnia	Insomnia	Insomnia	
Neuropathic pain, including diabetic neuropathy	Chronic pain conditions	Diabetic neuropathy	
Pruritus	Uremic pruritus	Fibromyalgia	
Interstitial cystitis	Prophylaxis of migraine	Interstitial cystitis (bladder pain syndrome)	
Postmenopausal hot flashes	Restless leg syndrome	Migraine prophylaxis	
Fibromyalgia	Complex regional pain syndrome	Postherpetic neuralgia	
Headache, including migraine prophylaxis		Sialorrhea	
Generalized tonic-clonic seizures			
Nausea and vomiting			
Irritable bowel syndrome			
Alcohol withdrawal			

**Table:** 

**Back pain, most common∗**	
Disorders of the cervical spine intervertebral discs	M50
Other disorders of the intervertebral discs	M51
Other back disorders not classified elsewhere	M53
Back pain	M54
**Back pain, other**	
Other deformities of the spine and back	M43
Ankylosing spondylitis	M45
Other inflammatory spondylopathies	M46
Spondylosis	M47
Other spondylopathies	M48
Spondylopathies in diseases classified elsewhere	M49
**Chronic pain**	
Other enthesopathies (diseases of peripheral ligament and muscle insertions)	M77
Osteoporosis with pathological fractures	M80
Biomechanical lesions, not elsewhere classified	M99
Endometriosis	N80
Ehlers-Danlos syndrome	Q796
Pain in the pelvis and pelvic floor	R102
Dislocation, sprain and strain of joints and ligaments at neck level	S13
Late symptoms of injury to cranial nerves	T90
Late symptoms of injury to the neck and trunk	T91
Late symptoms of injuries to the upper extremity	T92
Late symptoms of injuries to the lower extremity	T93
**Diabetes mellitus with complications∗**	
Diabetes mellitus type 1 - with neurological complications	E104
Diabetes mellitus type 1 - with multiple complications	E107
Diabetes mellitus type 1 - with unspecified complications	E108
Diabetes mellitus type 2 - with neurological complications	E114
Diabetes mellitus type 2 - with multiple complications	E117
Diabetes mellitus type 2 - with unspecified complications	E118
**Headaches∗**	
Migraine	G43
Other headache syndromes	G44
Headache	R51
**Neurological disorders∗**	
Benign tumor of the membranes of the central nervous system	D32
Benign tumor in brain and other parts of central nervous system	D33
Tumor of uncertain or unknown nature in the brain and other parts of the central nervous system	D43
Other basal ganglia disorders and movement disorders	G25
Multiple sclerosis	G35
Epilepsy	G40
Cerebral paresis	G80
Unilateral paralysis	G81
Parapares and tetrapares (paralysis of both lower extremities and paralysis of all four extremities)	G82
Other paralysis syndromes	G83
Other diseases of the spinal cord	G95
Late effects of cerebrovascular disease	I69
Spina bifida (split spine)	Q05
Gait disturbances and movement disorders	R26
**Neuropathies∗**	
Other spirochetal infections	A69
Shingles	B02
Diseases of the trigeminus nerve	G50
Diseases of other cerebral nerves	G52
Changes in cranial nerves in diseases classified elsewhere	G53
Diseases in brachial plexus	G54
Mononeuropathies of the upper extremity	G56
Mononeuropathies of the lower extremity	G57
Other mononeuropathies	G58
Hereditary and idiopathic neuropathy	G60
Inflammatory polyneuropathy	G61
Other polyneuropathies	G62
Polyneuropathy in diseases classified elsewhere	G63
Other diseases of the peripheral nervous system	G64
Damage to nerves and to the spinal cord in the neck region	S14
**Other diseases**	
Disrupted salivary gland function	K117
Irritable bowel	K58
Pruritus	L29
Interstitial cystitis	N301
Stress urinary incontinence	N393
Mixed urinary incontinence	N394C
Conditions associated with menopause	N951
Other specified medical conditions associated with menopause	N958
Medical condition associated with menopause, unspecified	N959
**Other arthritis**	
Seropositive rheumatoid arthritis	M05
Other forms of rheumatoid arthritis	M06
Psoriatic arthritis and arthritis in primary gastrointestinal diseases	M07
Juvenile arthritis	M08
Other specified arthropathies	M12
Other arthritis	M13
Arthropathies in other diseases classified elsewhere	M14
Other specified disorders in joints	M24
**Psychiatric conditions, most common∗**	
Depressive episode	F32
Other anxiety disorders	F41
Adjustment disorders and reaction to severe stress	F43
Non-organic sleep disorders	F51
Sleep disorders	G47
**Psychiatric conditions, other∗**	
Mental disorders and behavioral disorders caused by alcohol	F10
Manic episode	F30
Bipolar disease	F31
Recurrent depression	F33
Chronic mood disorders	F34
Other mood disorders	F38
Unspecified mood disorder	F39
Phobic syndrome	F40
Obsessive-compulsive disorder	F42
Dissociative disorders	F44
Somatoform disorders	F45
Neurotic, stress-related and somatoform syndromes	F48
Eating disorders	F50
Sexual dysfunction, not caused by organic disorder or disease	F52
Psychic disorders and behavioral disorders related to the puerperium, which are not classified elsewhere	F53
Behavioral disorders associated with physiological disturbances and physical factors	F55
Mild intellectual disability	F70
Moderate intellectual disability	F71
Severe intellectual disability	F72
Unspecified intellectual disability	F79
Hyperactivity disorders	F90
Conduct disorders of an outwardly directed type	F91
Mixed disorders of behavior and emotions	F92
Tics	F95
Behavioral disorders and emotional disorders with onset usually during childhood and adolescence	F98
Mental disorder not otherwise specified	F99
**Separate confounders**	
Other conditions of soft tissues not classified elsewhere∗	M79
Other joint diseases not classified elsewhere	M25
Pain and discomfort not classified elsewhere∗	R52
**Ulcers**	
Bed sores	L89
Leg ulcers, not classified elsewhere	L97
Other dermal and subcutaneous diseases, not classified elsewhere	L98

**Table:** 

Sex	Comorbidity	*Without* knee and/or hip OA		*With*knee and/or hip OA	
		Without comorbidity	With comorbidity	Without comorbidity	With comorbidity
Female	Back pain, most common	1.0	4.5	1.8	5.4
	Diabetes mellitus with complications	1.4	4.0	2.6	4.2
	Headaches	1.3	3.6	2.6	3.9
	Other conditions of soft tissues not classified elsewhere	1.0	5.1	1.8	5.5
	Neurological disorders	1.3	5.1	2.5	5.2
	Neuropathies	1.3	4.4	2.4	5.8
	Psychiatric conditions, most common	0.7	3.7	1.7	4.7
	Psychiatric conditions, other	1.2	6.3	2.3	8.5
	Pain and discomfort not classified elsewhere	1.0	4.5	1.9	5.1
Male	Back pain, most common	0.5	3.2	1.0	3.7
	Diabetes mellitus with complications	0.7	2.7	1.4	2.8
	Headaches	0.7	2.4	1.4	3.2
	Other conditions of soft tissues not classified elsewhere	0.7	2.5	1.2	3.3
	Neurological disorders	0.7	3.9	1.2	4.7
	Neuropathies	0.7	4.0	1.2	4.9
	Psychiatric conditions, most common	0.4	2.8	0.9	3.6
	Psychiatric conditions, other	0.6	3.6	1.3	4.7
	Pain and discomfort not classified elsewhere	0.6	3.3	1.0	3.7

**Figure:**
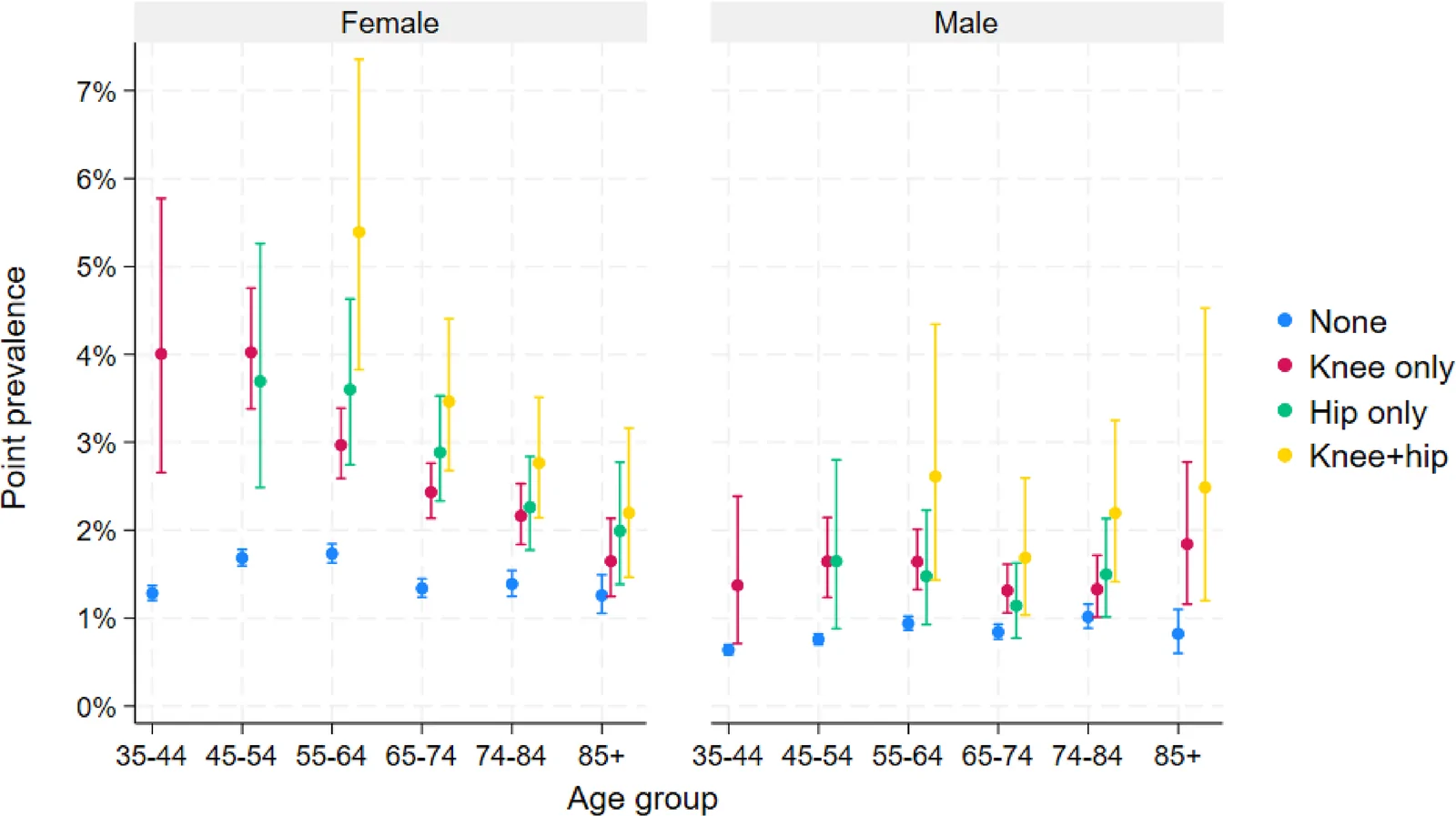


**Table:** 

	OA site	Risk ratio	95% CI	Risk difference	95% CI
Demographics	Knee	1.64	1.46–1.86	0.0052	0.0036–0.0067
Demographics	Hip	1.46	1.20–1.79	0.0037	0.0014–0.0061
Demographics	Knee + hip	2.20	1.72–2.81	0.0096	0.0054–0.0138
Demographics + comorbidities	Knee	1.04	0.92–1.19	0.0004	−0.0007–0.0014
Demographics + comorbidities	Hip	0.87	0.70–1.07	−0.0011	−0.0026–0.0005
Demographics + comorbidities	Knee + hip	0.96	0.73–1.25	−0.0004	−0.0024–0.0017

**Table:** 

	RR	95%CI	RD	95%CI
Knee or hip OA but no TKR or THR	1.02	0.90–1.15	0.0001	−0.0009–0.0012
Knee or hip OA and TKR or THR	0.92	0.76–1.12	−0.0006	−0.0021–0.0008

**Figure:**
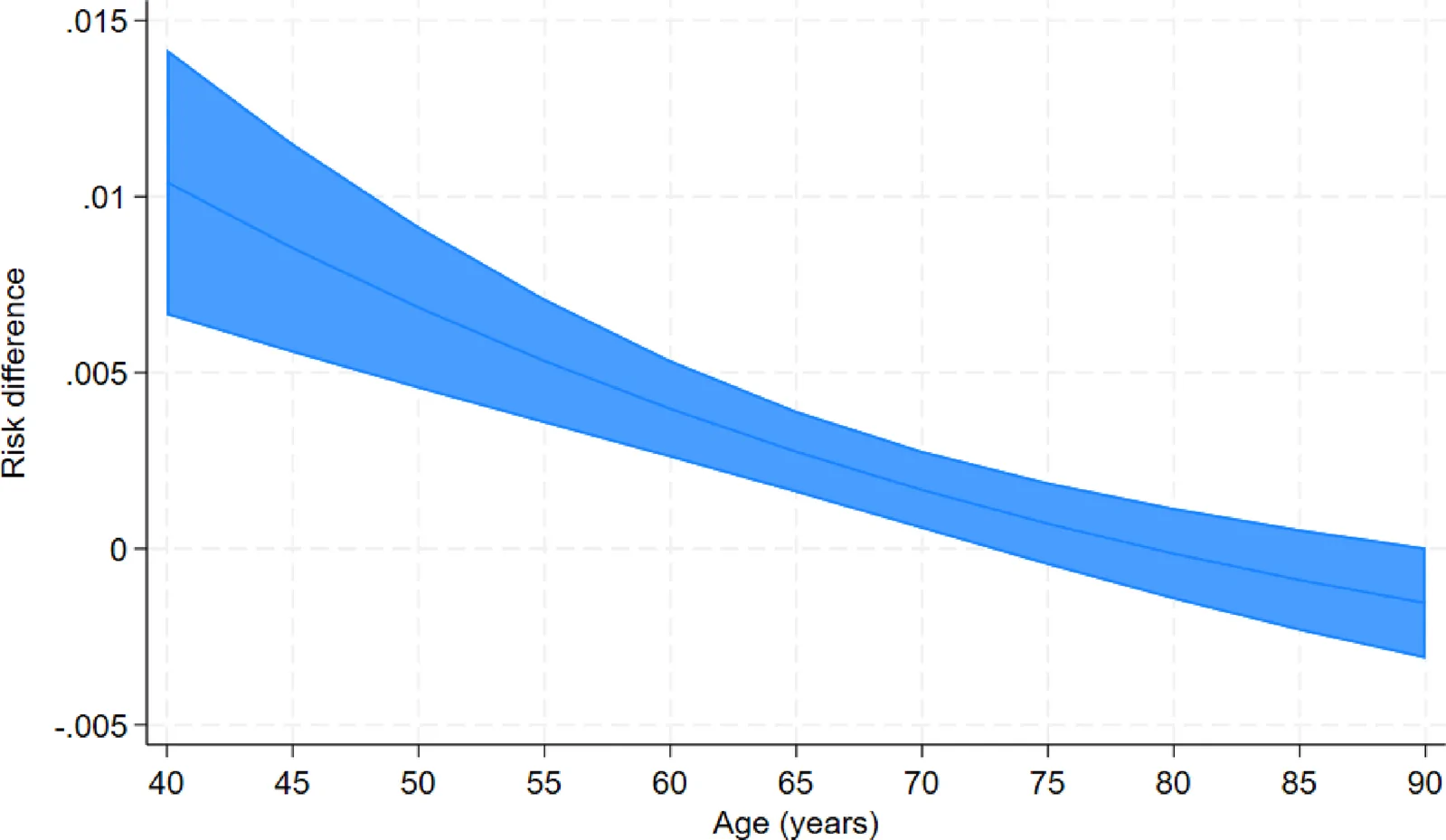


**Table:** 

	Age	Knee OA				Hip OA				Knee and hip OA			
		RR	95%CI	RD	95%CI	RR	95%CI	RD	95%CI	RR	95%CI	RD	95%CI
Demographics	40	2.89	2.43–3.43	0.0303	0.0228–0.0378	3.31	2.46–4.44	0.0367	0.0220–0.0515	4.79	3.02–7.61	0.0589	0.0268–0.0910
Demographics	50	2.40	2.13–2.71	0.0214	0.0172–0.0256	2.74	2.22–3.40	0.0264	0.0179–0.0350	3.77	2.69–5.29	0.0414	0.0233–0.0594
Demographics	60	2.00	1.84–2.17	0.0144	0.0121–0.0167	2.28	1.97–2.64	0.0184	0.0137–0.0230	2.97	2.37–3.72	0.0281	0.0189–0.0372
Demographics	70	1.66	1.53–1.81	0.0090	0.0073–0.0108	1.89	1.67–2.14	0.0121	0.0091–0.0151	2.34	2.01–2.73	0.0181	0.0135–0.0227
Demographics	80	1.38	1.22–1.56	0.0049	0.0029–0.0070	1.57	1.33–1.85	0.0073	0.0042–0.0105	1.84	1.54–2.21	0.0108	0.0068–0.0149
Demographics	90	1.15	0.97–1.37	0.0018	−0.0005–0.0042	1.30	1.03–1.65	0.0037	0.0000–0.0073	1.45	1.10–1.92	0.0055	0.0007–0.0103
Demographics + comorbidities	40	1.55	1.30–1.85	0.0093	0.0049–0.0136	1.72	1.27–2.34	0.0120	0.0037–0.0203	1.99	1.23–3.22	0.0162	0.0015–0.0309
Demographics + comorbidities	50	1.39	1.23–1.57	0.0061	0.0035–0.0088	1.52	1.22–1.90	0.0081	0.0030–0.0133	1.64	1.15–2.33	0.0099	0.0013–0.0186
Demographics + comorbidities	60	1.24	1.14–1.36	0.0036	0.0020–0.0052	1.34	1.15–1.57	0.0050	0.0021–0.0080	1.35	1.06–1.71	0.0051	0.0005–0.0097
Demographics + comorbidities	70	1.11	1.02–1.22	0.0016	0.0003–0.0029	1.18	1.04–1.35	0.0025	0.0005–0.0046	1.11	0.94–1.30	0.0015	−0.0010–0.0040
Demographics + comorbidities	80	1.00	0.88–1.13	−0.0001	−0.0017–0.0016	1.04	0.88–1.23	0.0006	−0.0017–0.0028	0.91	0.76–1.10	−0.0011	−0.0034–0.0011
Demographics + comorbidities	90	0.89	0.75–1.06	−0.0013	−0.0033–0.0006	0.92	0.72–1.17	−0.0010	−0.0037–0.0018	0.75	0.56–1.00	−0.0031	−0.0058–−0.0003

**Table:** 

	Age	RR	95%CI		RD	95%CI
Knee or hip OA but no TKR or THR	40	1.48	1.25	1.74	0.0080	0.0041–0.0120
Knee or hip OA but no TKR or THR	50	1.32	1.17	1.49	0.0051	0.0027–0.0075
Knee or hip OA but no TKR or THR	60	1.18	1.08	1.29	0.0027	0.0012–0.0042
Knee or hip OA but no TKR or THR	70	1.05	0.97	1.15	0.0008	−0.0005–0.0020
Knee or hip OA but no TKR or THR	80	0.94	0.84	1.0	−0.0008	−0.0023–0.0007
Knee or hip OA but no TKR or THR	90	0.84	0.72	0.99	−0.0019	−0.0037 to −0.0001
Knee or hip OA and TKR or THR	40	2.63	1.92	3.59	0.0257	0.0139–0.0375
Knee or hip OA and TKR or THR	50	2.09	1.67	2.62	0.0166	0.0098–0.0233
Knee or hip OA and TKR or THR	60	1.66	1.43	1.93	0.0096	0.0062–0.0131
Knee or hip OA and TKR or THR	70	1.32	1.18	1.48	0.0045	0.0025–0.0064
Knee or hip OA and TKR or THR	80	1.05	0.91	1.22	0.0007	−0.0013–0.0027
Knee or hip OA and TKR or THR	90	0.84	0.67	1.04	−0.0020	−0.0043–0.0003
